# Efficacy and safety of massage therapy for chronic atrophic gastritis: A protocol for systematic review and meta-analysis

**DOI:** 10.1097/MD.0000000000023347

**Published:** 2020-11-20

**Authors:** Ke-Lin Zhou, Shuo Dong, Sheng Guo, Xiao-Hui Dai, Jing-Yi Yang, Yang Liu, Bao-Lai Mi, Shao-Wei Wang, Guo-Bing Fu, Pei-Dong Wei

**Affiliations:** a Dongfang Hospital of Beijing University of Chinese Medicine; b School of Traditional Chinese Medicine, Beijing University of Chinese Medicine, Beijing, China.

**Keywords:** chronic atrophic gastritis, complementary and alternative medicine, massage therapy, protocol

## Abstract

**Background:**

Chronic atrophic gastritis (CAG) is an established precursor of gastric carcinoma with high prevalence worldwide. It is a typical complex gastro-intestinal disease with multiple influence factors, of which exact mechanisms remain unelucidated. Therefore, an ideal strategy to relieve CAG is urgently needed. In recent years, massage therapy has been increasingly accepted by CAG patients due to its lower costs, fewer unwanted side effects and safety for clinical use. In this systematic review, we aim to evaluate the effectiveness and safety of massage therapy for patients with chronic atrophic gastritis.

**Methods:**

We will search the following electronic databases for randomized controlled trials to evaluate the effectiveness and safety of massage therapy in treating chronic atrophic gastritis: Wanfang and Pubmed Database, China National Knowledge Infrastructure Database, Cochrane Central register of controlled trials, Cumulative Index of Nursing and Allied Health Literature, and Excerpta Medica database. Each database will be searched from inception to September 2020. The entire process will include study selection, data extraction, risk of bias assessment, and meta-analyses.

**Result:**

This proposed study will evaluate the effectiveness and safety of massage therapy for patients with chronic atrophic gastritis. The outcomes will include changes in CAG relief and adverse effect.

**Conclusion:**

This proposed systematic review will evaluate the existing evidence on the effectiveness and safety of massage therapy for patients with chronic atrophic gastritis.

**Dissemination and ethics:**

The results of this review will be disseminated through peer-reviewed publication. Because all of the data used in this systematic review and meta-analysis has been published, this review does not require ethical approval. Furthermore, all data will be analyzed anonymously during the review process.

## 1 Introduction

Chronic atrophic gastritis (CAG), a kind of functional gastrointestinal disease, has various clinical symptoms mainly include fullness, epigastric pain, anorexia, belching, and other nonspecific symptoms.^[1,2]^ It is characterized by atrophic gastric mucosa, reduced gastric acid secretion and pathological changeable epithelium along with intestine epithelium metaplasia.^[3,4]^ Chronic atrophic gastritis (CAG) is an established pre-cancerous lesion of intestinal type gastric cancer which is ranked fifth in the cancer incidence and is the third leading cause of cancer death reported in the global cancer statistics 2018.^[5–7]^ The incidence of CAG arises from repeated lesions of gastric superficial mucosa induced by multiple endogenous and exogenous factors such as emotional stress, physicochemical factors, and microbial infection.^[8]^ The potential mechanisms include immune-mediated inflammation, mucosal gland atrophy, increased serum auto antibodies to gastric parietal cells and/or intrinsic factors, hypochlorhydria, vitamin B12 deficiency and, in some cases, neurological symptoms and diffuse metaplasia.^[9–11]^ At present, the clinical treatments of CAG are mainly relying on the synthetic medicine such as vitamin B12 and vitacoenzyme,^[12]^ which remain unsatisfying due to its long course of application, recurrent episodes, invasive and adverse effects.^[13]^ Therefore, an ideal strategy to relieve CAG is urgently needed. In recent years, traditional Chinese medicine (TCM) has been increasingly accepted by CAG patients due to its dual functions of treatment and coordinating, widely available and fewer side effects.^[14,15]^

Massage therapy, one of the most popular complementary and alternative therapies, have been used for thousands of years in China. Currently, they are increasingly used because of their lower costs and safety for clinical use.^[16]^

This review aims to systematically review all randomized controlled trials (RCTs) to assess the effectiveness and safety of massage treatment for patients with chronic atrophic gastritis.

## 2 Materials and methods

This systematic review protocol has been registered on OSF on October 21, 2020 (Registration number: DOI 10.17605/OSF.IO/83KXY). The protocol follows the Cochrane handbook for systematic reviews of interventions and the preferred reporting items for systematic reviews and meta-analysis protocol statement guidelines.^[17]^ We will describe the changes in our full review if needed.

## 3 Inclusion criteria for study selection

### 3.1 Type of studies

This review will include clinical RCTs of clinical massage therapy for chronic atrophic gastritis (CAG) patients without any language or publication status restrictions. Non-RCTs, quasi-RCTs, case series, case reports, crossover studies, uncontrolled trials, and laboratory studies will not be included.

### 3.2 Type of participants

Participants who were diagnosed with CAG according to related guidelines or consensus. All included participants in this review regardless of their age, race, and gender. Pregnant and lactating women will be excluded.

### 3.3 Type of interventions

Interventions will include any type of clinically performed massage for improvement of chronic atrophic gastritis. This will include Chinese massage, Japanese massage, Thai massage, Swedish massage, Tuina, Shiatsu, remedial massage, general massage, acupressure, reflexology, manual lymphatic drainage. Studies of CAG combined with other interventions such as acupuncture, herbal medicines, qigong and yoga will be considered for exclusion.

Control: no intervention, treatments other than massage (e.g., usual or standard care, placebo, wait-list controls).

### 3.4 Type of outcome measures

#### 3.4.1 Main outcome(s)

The primary outcome at the end of treatment or at maximal follow-up is the clinical effective rate, which is categorized as cure, markedly effective, effective, or ineffective according to clinical symptoms, degree of gastric mucosal lesion under gastroscopy and athological changes of gastric mucosa, etc.

#### 3.4.2 Additional outcome(s)

The secondary outcomes will include clearance of HP infection, quality of life (SF-36), symptom scores (stomachache, stomach distention, belching and acid reflux, etc), comparison of therapeutic effects under gastroscope, and comparison of curative effect of pathological tissue, etc.

## 4 Search methods for the identification of studies

### 4.1 Electronic searches

We will search the following electronic bibliographic databases for relevant trials:

China National Knowledge Infrastructure Database (from 1979 to present);
Wanfang Database (from 1990 to present);
Pubmed Database (from 2000 to present);
Cochrane Central Register of Controlled Trials (from 2000 to present);
Cumulative Index of Nursing and Allied Health Literature, (from 1937 to present);
Excerpta Medica database (from 1947 to present);
Ovid Medical Literature Analysis and Retrieval System Online (from 1946 to present);
In addition, clinical trial registries, like the Chinese Clinical Trial Registry (ChiCTR), the Netherlands National Trial Register (NTR) and ClinicalTrials.gov, will be searched for ongoing trials with unpublished data.
There will be no language restrictions.

### 4.2 Data collection and analysis

#### 4.2.1 Study identification

We will use EndNote X9 software to manage the records of searched electronic databases. The initial selection will involve scanning of the titles and abstracts of the retrieved studies. The full text of relevant studies will then be reviewed for study inclusion, in accordance with the inclusion criteria, by 2 authors (KLZ and SD). Potentially relevant articles will be reviewed independently by 2 authors to determine if they meet the prespecified criteria. Any disagreement between authors will be resolved by consensus with a third author. The study selection procedure will follow and be recorded in the preferred reporting items for systematic reviews and meta-analysis flow chart. All the evidence will be assessed by the grading of recommendations assessment, development, and evaluation.

#### 4.2.2 Data extraction and management

According to the inclusion criteria, a standard data collection form will be made before data extraction. The following data will be extracted by 2 authors (KLZ and SD):

*General information*: research identification, publication year, the title of the study, first author;
*Study methods*: study design, sample size, randomization method, allocation concealment, blinding, incomplete report or selecting report, other sources of bias;
*Participants*: inclusion and exclusion criteria;
*Intervention*: motion details, treatment duration, and frequency;
*Control*: type of control methods, motion details, treatment duration, and frequency;
*Outcomes*: included outcome measures.

#### 4.2.3 Risk of bias assessment

The risk of bias in included studies will be assessed independently by 2 reviewers (KLZ and SD) using the Cochrane Risk of Bias Tool, with any disagreements resolved by consensus or by discussion with a third reviewer. All judgments will be fully described, and the conclusions will be presented in the risk of bias figures and will be incorporated into the interpretation of review findings, by means of sensitivity analysis. The risk of bias of each domain will be graded as adequate, unclear, or inadequate. We intend to use the concealment of allocation grading in investigation of any heterogeneity and in sensitivity analysis. Other aspects of study quality including the extent of blinding (if appropriate), losses to follow up, non-compliance, whether the outcome assessment was standardized, and whether an intention to treat analysis was undertaken, will be presented in the risk of bias table describing the included studies and will provide a context for discussing the reliability of the results.

#### 4.2.4 Data analysis

We will use Stata Software [Computer program] (Version 15.1) to process the meta-analysis. Weighted mean difference will be used for continuous variable data, and the combined statistical effects of these 2 are combined. The X^2^ test will be adopted to analyze whether there is heterogeneity in each of the included research questions. I^2^ > 50% is a criterion for significant judgment. The fixed effect model is adopted if I^2^ ≤ 50%, which is considered to have homogeneity between the studies. The random effect model is adopted if I^2^ > 50%, which is considered to have heterogeneity among the studies. The effect size is expressed as 95% confidence interval, and *P* < .05 is considered to be statistically significant.

***Sensitivity analyses:*** heterogeneity may be due to the presence of 1 or more outlier studies with results that conflict with the rest of the studies. We will perform sensitivity analyses excluding outlier studies. In addition, we plan to perform sensitivity analysis to explore the influence of trial quality on effect estimates. The quality components of methodology include adequacy of generation of allocation sequence, concealment of allocation, and the use of intention-to-treat analysis.

***Meta-regression analyses:*** if data permits, we will perform the meta-regression analyses.

#### 4.2.5 Publication bias

If sufficient number of trials (more than 10 trials) are found, we will generate funnel plots (effect size against standard error) to investigate publication bias.

#### 4.2.6 Ethics and dissemination

The data used in this systematic review will be collected from published studies. Based on this, the study does not require ethical approval.

## Acknowledgments

The authors would like to thank all the researchers in our working group.

## Author contributions

KLZ, SD, PDW contributed on methodology and are the guarantors of the review.

KLZ, SD, SG and PDW contributed on data search, analysis, and statistics.

**Conceptualization:** Kelin Zhou, Shuo Dong, Xiaohui Dai.

**Data curation:** Ke-Lin Zhou, Shuo Dong, Xiao-Hui Dai, Jing-Yi Yang, Yang Liu, Bao-Lai Mi, Shao-Wei Wang.

**Formal analysis:** Sheng Guo, Xiaohui Dai.

**Funding acquisition:** Pei-Dong Wei.

**Investigation:** Jingyi Yang.

**Methodology:** Ke-Lin Zhou, Shuo Dong, Pei-Dong Wei.

**Resources:** Pei-Dong Wei, Sheng Guo.

**Software:** Yang Liu, Baolai Mi, Shaowei Wang Ke-Lin Zhou.

**Supervision:** Guo-Bing Fu, Pei-Dong Wei.

**Writing – original draft:** Ke-Lin Zhou, Shuo Dong.

**Writing – review & editing:** Guo-Bing Fu, Sheng Guo, Pei-Dong Wei.
